# Successful abdominal wall closure following collagen-based artificial dermis induced epithelialization for giant omphalocele: A case report

**DOI:** 10.1016/j.ijscr.2020.09.096

**Published:** 2020-09-17

**Authors:** Masaki Horiike, Tomohiro Kitada, Kenji Santo, Takuro Hashimoto, Onishi Satoshi

**Affiliations:** a Department of Pediatric Surgery, Japanese Red Cross Society, Wakayama Medical Center, 4-20 Komatsubara-dori, Wakayama, Wakayama, 640-8558, Japan; b Department of Surgery, Matsushitakai, Shiraniwa Hospital, 6-10-1, Shiraniwa-dai, Ikoma, Nara, 630-0136, Japan; c Department of Pediatric Surgery, Osaka City University Graduate School of Medicine, 1-4-3, Asahi-machi, Abeno-ku, Osaka, 545-8585, Japan; d Department of Pediatrics, Osaka City University Graduate School of Medicine, 1-4-3, Asahi-machi, Abeno-ku, Osaka, 545-8585, Japan

**Keywords:** GO, giant omphalocele, NPWT, negative pressure wound therapy, SWR, small size wound retractor, CAD, collagen-based artificial dermis, Giant omphalocele, Silo infection, Jejunal perforation, Negative pressure wound therapy, Collagen-based artificial dermis, Case report

## Abstract

**Introduction:**

A giant omphalocele (GO) with marked viscero-abdominal disproportion is associated with surgical difficulty and higher morbidity and mortality rates. Despite various treatment strategies, no consensus exists on optimal GO management. We report the clinical course of a neonate with a GO who was successfully treated with abdominal-wall closure through the novel application of collagen-based artificial dermis (CAD) for epithelization.

**Presentation of case:**

A female neonate (estimated gestational age, 38 weeks; birthweight, 3.047 kg) with a GO where most viscera, including the liver, were completely herniated. G-band analysis showed no chromosomal abnormality and normal karyotype. Conventional silo formation was attempted, but incomplete silo was formed due to adhesion between the portal vein and fascia, and repatriation of the herniated viscera had not progressed. A new silo was formed using biomaterial, but it was infected and removed. Abdominal wall epithelialization using NPWT was attempted again but was interrupted by the occurrence of jejunal perforation. After incising the epithelialized part of the abdominal wall and repairing the perforated jejunum, the GO was covered and fixed using CAD. Epithelialization progressed well, and she was discharged on day 328.

**Discussion:**

In this case, the major therapeutic challenges (including formation of an incomplete silo, silo infection, and jejunal perforation) were overcome with conventional treatment except for epithelialization of the abdominal wall, which was achieved by using CAD.

**Conclusion:**

The treatment with CAD for epithelialization can be considered in cases where it is extremely difficult to return the viscera in conventional management.

## Highlights

• A giant omphalocele (GO) is related to higher rates of morbidity and mortality.
• No consensus exists on optimal GO management, which may be surgically challenging.
• We report the successful GO management of a neonate with numerous complications.
• We applied a collagen-based artificial dermis for epithelization as a new treatment.

## 1 Introduction

Omphalocele is a congenital abdominal wall defect that usually results in herniation of the abdominal viscera within a sac or peritoneum and amnion. An omphalocele occurs in 1 in 4,000–6,000 live births [1]. A giant omphalocele (GO), which lacks a strict clinical definition, is commonly described as a fascial defect > 0.05 m or which contains more than 50 or 75% of the liver within the sac and occurs in 1 in 10,000 live births [2]. A GO with marked viscero-abdominal disproportion is associated with higher rates of morbidity and mortality [3]. Hence, it often represents a major surgical challenge. Although various treatment strategies such as initial operative closure, staged closure, and several delayed closures exist, there is no consensus on the optimal management for a GO [4].

We present our experience with a neonate with GO where all abdominal organs had herniated except the duodenum, rectum, kidneys, uterus, and ovaries. There was a high degree of viscero-abdominal disproportion. However, we succeeded in closing the abdominal wall by epithelialization, utilizing a combination of previously reported negative pressure wound therapy (NPWT) [5] and collagen-based artificial dermis as a novel treatment method. The complex clinical course and the complications encountered are elaborated here. This work was reported in line with the SCARE 2018 criteria [6]. Research Registry UIN is 5920.

## 2 Presentation of case

**Fig. 1:**
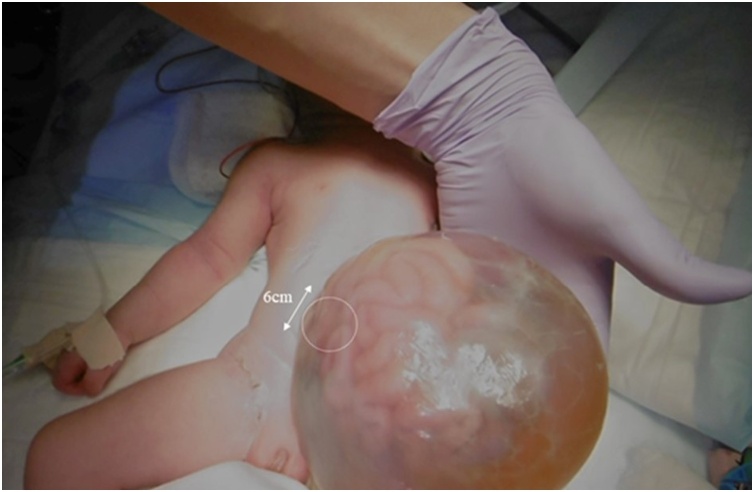
Giant omphalocele (GO) findings immediately after birth. A female neonate was born at 38 weeks estimated gestational age with a birthweight of 3047 g. She had a GO with a fascial defect measuring 6 × 6 cm, and no abnormalities were found other than the GO.

A Japanese female neonate was born at an estimated gestational age of 38 weeks with a birthweight of 3.047 kg. She had an intact GO with a fascial defect measuring 0.06 × 0.06 m (Fig. 1). No other associated abnormalities were found on screening including postnatal echocardiography. G-band analysis showed no chromosomal abnormality and normal karyotype. As the sac containing several protuberant viscera was large, pedunculated, and unsupported, the sac rolled over. The hemodynamics changed and became unstable, leading to low blood pressure.

**Fig. 2:**
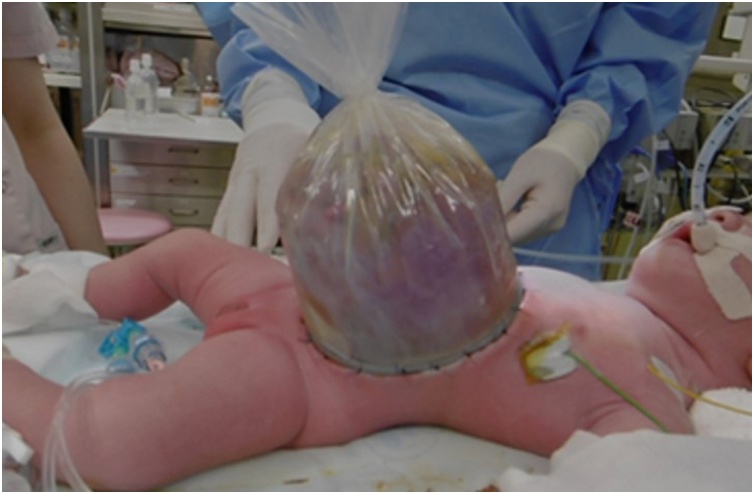
Findings fixing the small size wound retractor (SWR) to the abdominal wall. Since the hepatic veins firmly adhered to the cranial fascia of the abdominal wall defect and the SWR could not be inserted the fascial defect, the SWR was fixed to the abdominal wall.

**Fig. 3:**
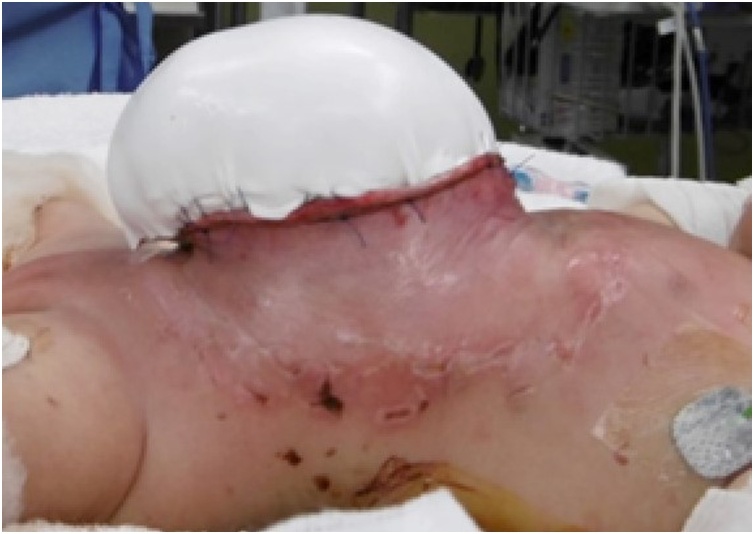
Findings fixing a biomaterial soft tissue patch to the fascia around the abdominal wall defect. A biomaterial soft tissue patch was sutured to the fascia around the abdominal wall defect to form a new silo after removing the small size wound retractor and amniotic membrane.

On the day of birth, a silo was formed using a small size wound retractor (SWR) (Alexis Wound Retractor small size, Applied Medical Resources Corporation, Rancho Santa Margarita, CA, USA) to support the large sac. Since the hepatic veins were firmly adherent to the cranial end of the rectus fascia of the abdominal wall defect, the SWR could not be inserted through the fascial defect. Hence, the SWR was fixed to the abdominal wall (Fig. 2). The silo was crimped daily, but repatriation of the herniated viscera had not progressed. The abdominal wall to which SWR was fixed was incised because numerous injuries had resulted from the excessive tension that had been applied to the fixed parts. Therefore, biomaterial (GORE-TEX® Soft tissue patch, W. L. Gore & Associates, Inc., Newark, NJ, USA) was sutured to each fascia to form a new silo after removing the SWR and sac (Fig. 3). In addition, a sterile dressing of silver (AQUACEL®Ag Extra TM, ConvaTec, England & Wales) was attached to prevent infection in the silo. However, by day 51 the silo had become infected and resulted in bacteremia.

The infected silo was removed quickly, but a large quantity of pus was noted adhering to the surface of the protruding viscera. A new silo was reformed using an SWR, and the infected surface was washed with saline daily.

**Fig. 4:**
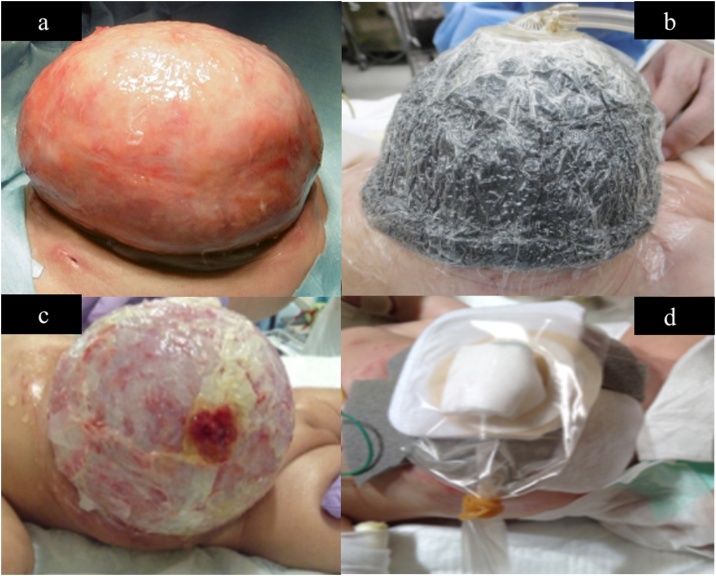
a–d: Findings from the silo infection control to introducing the negative pressure wound therapy (NPWT) device to the jejunal perforation and taking countermeasures.

By day 65, the infection was under control, and a fibrous capsule had formed on the surface of the viscera and had stabilized them (Fig. 4a). On the other hand, the abdominal wall had become thin and fragile, making it difficult to stretch it and close the abdominal wall. Hence, we decided that it would be impossible to close the wall by reducing the silo contents and considered the idea of epithelializing the abdominal wall defect, leading to closure. We utilized the NPWT method: non-adherent wound dressings (Mepitel®, Molnlycke Healthcare, Gothenburg, Sweden), WHITEFORM® dressings, and GRANUFORM® dressings were applied in this order, and then a clear film was applied to the entire omphalocele. The apex was cut into 1 cm squares and connected to the negative pressure system. The system was set at -25 mmHg (Fig. 4b). Using this method, epithelization proceeded smoothly for a while. On day 71, it was discovered that a part of the epithelializing capsule had ruptured, and the jejunum had perforated (Fig. 4c).

Since the new epithelium that had formed was incomplete and fragile, and the perforated jejunum had adhered to the capsule, intestinal fluid did not leak into the abdominal cavity. Thus, the perforated jejunum was managed as a natural intestinal fistula.

**Fig. 5:**
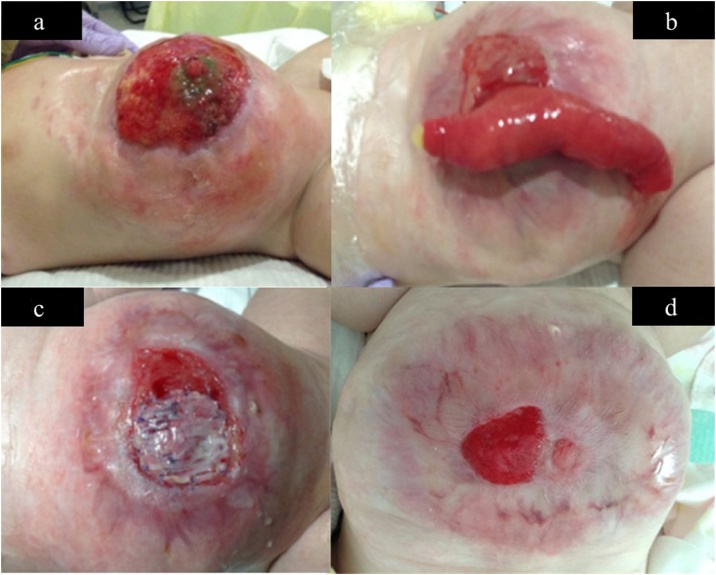
a–d: Findings from incomplete epithelialization to repairing the perforated jejunum and completing the epithelialization using a collagen-based artificial dermis.

As a result of absorbing the intestinal fluid discharge with gauze, and protecting the epithelial coating with an absorbent foam dressing (Mepilex®, Mölnlycke Health Care AB. Sweden) (Fig. 4d), re-epithelialization of the capsule progressed smoothly (Fig. 5a).

By day 178, the epithelial layer had advanced to the periphery of the natural intestinal fistula (Fig. 5b), and hence, an incision was made along the epithelialization area, and the intestinal fistula was drawn out and repaired. On the other hand, it was impossible to close the incision area with sutures because the abdominal wall was thin and fragile. Therefore, a collagen-based artificial dermis (Terudermis®, Olympus Terumo Biomaterials Corp, Tokyo, Japan) was cut according to the size of the incision area and fixed by suturing (Fig. 5c).

By day 247, epithelialization of the abdominal wall was almost complete (Fig. 5d), and on day 328, the patient was discharged from our hospital. She regularly visits our pediatric surgery outpatient department and was found to be well at the latest follow up, 1 year later, without postoperative complications.

## 3 Discussion

Although multiple management strategies have been developed for a GO, we failed to achieve abdominal wall closure with those conventional strategies in this case [[1], [2], [3], [4]].

Our patient presented with three major therapeutic challenges. One was the formation of an incomplete silo. Since our patient had a completely herniated liver, the pedicled nature resulted in vena caval compression leading to low blood pressure. Harjai [7] succeeded in viscera repatriation in a case of a GO that contained a completely herniated liver by suturing a silastic silo circumferentially over the abdominal wall defect and suspending the apex to stabilize the liver. In our case, the hepatic veins that were adherent to the fascia obstructed complete silo formation, as they interfered with the sutures that were used to fix the silo to the abdominal wall, and prevented viscera repatriation.

The second was silo infection. Pampaloni [8] reported that they successfully treated five newborns with large congenital abdominal wall defects, in whom fascial closure could not be accomplished by primary repair with a biomaterial (GORE-TEX® soft tissue patch). We used a Gore-Tex patch to form the silo, and because skin closure was impossible, we attached a sterile dressing of silver (AQUACEL®Ag Extra TM) around the silo to prevent infection.

Although the silo made of biomaterial (GORE-TEX® soft tissue patch) was fixed to the fascia and provided excellent support to the protuberant viscera, silo infection had led to the potential failure of viscera repatriation. Because drug-resistant bacteria emerged, we removed the silo instead of providing antimicrobial treatment.

The third was jejunal perforation. The strategy was changed from viscera repatriation to epithelialization of the abdominal wall utilizing the NPWT method, following which, enteral nutrition was started. However, she had to be maintained nil-per-oral again because of the occurrence of jejunal perforation. NPWT is a safe and effective treatment for GO, but NPWT may cause intestinal perforation if the GO capsule is fragile. The intestinal drainage caused delayed epithelialization, but we were eventually able to achieve closure of the jejunal perforation by swabbing the drainage with gauze and dressing the abdominal wall to minimize the delay in epithelialization. Our experience shows that jejunal perforation can be overcome, although it is a relatively rare complication of GO.

We were persistent in addressing the above-mentioned therapeutic challenges. We were able to achieve abdominal wall closure by introducing the novel idea of using artificial dermis to close the abdominal wall by epithelialization.

The abdominal wall closed by this method had a defect in the fascia, for which we have planned reconstruction of the abdominal wall in the future in two phases once the baby achieves physical maturity.

## 4 Conclusion

Although two-stage surgery is required, treatment with collagen-based artificial dermis for epithelization can be considered in cases where it is extremely difficult to return the viscera to the abdomen or in cases where gastrointestinal perforation leads to difficulties in conventional management.

## Declaration of Competing Interest

The authors report no declarations of interest.

## Funding

All authors declare that they have no funding source.

## Ethical approval

This submission is a case report about surgical technique, not the manuscript reporting studies involving human participants, human data or human tissue.

In this case, ethical approval has been exempted by our institution.

## Consent

Written informed consent was obtained from the patient for publication of this case report and accompanying images. A copy of the written consent is available for review by the Editor-in-Chief of this journal on request.

## Author contribution

Masaki Horiike (MH) made the conception and design of this case report. Authors other than MH contributed to the collection, analysis, and interpretation of the data. MH wrote the draft manuscript, and other authors performed the critical revision of the manuscript.All authors gave final approval of the version to be published. MH has overall responsibility and guarantees the scientific integrity.

## Registration of research studies

1. Name of the registry: Masaki Horiike.
2. Unique identifying number or registration ID: Researchregistry5920.
3. Hyperlink to your specific registration (must be publicly accessible and will be checked): https://www.wakayama-med.jrc.or.jp/department/shonigeka/.

## Guarantor

MH has overall responsibility and guarantees the scientific integrity.

## Provenance and peer review

Not commissioned, externally peer-reviewed.

## References

[1] Bauman B., Stephens D., Gershone H., Bongiorno C., Osterholm E., Acton R. (2016). Management of giant omphaloceles. A systematic review of methods of staged surgical vs nonoperative delayed closure. J. Pediatr. Surg..

[2] McNair C., Hawes J., Urquaht H. (2006). Caring for the newborn with an omphalocele. Neonat. Netw..

[3] Wagner J.P., Cusick R.A. (2019). Paint and wait management of giant omphaloceles. Semin. Pediatr. Surg..

[4] Oquendo M., Agrawal V., Reyna R., Patel H.I., Emran M.A., Almond P.S. (2015). Silver-impregnated hydrofiber dressing followed by delayed surgical closure for management of infants born with giant omphaloceles. J. Pediatr. Surg..

[5] Aldridge B., Ladd A.P., Kepple J., Wingle T., Ring C., Kokoska E.R. (2016). Negative pressure wound therapy for initial management of giant omphalocele. Am. J. Surg..

[6] Agha R.A., Borrelli M.R., Farwana R., Koshy K., Fowler A.J., Orgill D.P. (2018). The SCARE 2018 statement: updating consensus surgical CAse REport (SCARE) guidelines. Int. J. Surg..

[7] Harjai M.M., Bhargava P., Sharma A., Saxena A., Singh Y. (2000). Repair of a giant omphalocele by a modified technique. Pediatr. Surg. Int..

[8] Pampaloni F., Pampaloni A., Noccioli B., Mattei R. (1998). Use of a Gore-Tex patch in the primary repair of congenital defects of the anterior abdominal wall. Pediatr. Med. Chir..

